# Associations Between Controlling Nutritional Status and Allostatic Load With Heart Failure Across Different Depressive States: A Cross-Sectional Study Using NHANES 2005–2018 Data

**DOI:** 10.31083/RCM45879

**Published:** 2026-02-03

**Authors:** Lai Li, Yujia Zhai, Aijun Liu, Junwu Su

**Affiliations:** ^1^Department of Cardiac Surgery, Beijing Anzhen Hospital, Capital Medical University, 100029 Beijing, China; ^2^Department of Wuhan Mental Health Center, Jianghan University, 430000 Wuhan, Hubei, China

**Keywords:** heart failure, CONUT, AL, depression

## Abstract

**Background:**

The controlling nutritional status (CONUT) and allostatic load (AL) indices indicate significant correlations with heart failure (HF). Given that depressive status associated with metabolic dysregulation may influence these associations, this research aimed to explore whether depressive status modulates the associations between these two indices and HF.

**Methods:**

Data were analyzed from 4632 participants aged ≥20 years in the National Health and Nutrition Examination Survey (NHANES), 2005–2018. After applying weighting (WTINT2YR) to the included data, samples with missing data and those without weighted processing were excluded. Binary logistic regression analysis was then employed to investigate the relationships between CONUT, AL, and HF. Subgroup analysis was performed with depressive status as a stratifying factor, and a restricted cubic spline (RCS) model was used to investigate the presence of linear or non-linear relationships between the two clinical indices and HF. Receiver operating characteristic (ROC) curves, net reclassification improvement (NRI), and integrated discrimination improvement (IDI) were employed to evaluate the predictive performance of the different models for HF.

**Results:**

Both CONUT and AL were positively correlated with HF in Model 1 (CONUT: odds ratio (OR) = 1.43, 95% confidence interval (CI): 1.25–1.63, *p* < 0.001; AL: OR = 1.23, 95% CI: 1.14–1.32, *p* < 0.001) and Model 2 (CONUT: OR = 1.29, 95% CI: 1.12–1.48, *p* < 0.001; AL: OR = 1.14, 95% CI: 1.05–1.24, *p *= 0.002). Depressive status was shown to moderate the positive association between CONUT and HF (*p* for interaction = 0.035). AL was associated with HF in the depressive subgroup (area under the curve (AUC) = 0.6048, 95% CI: 0.5162–0.6934), indicating limited predictive performance of the model. The NRI and IDI values revealed no significant difference in the predictive performance of CONUT and AL in Model 4.

**Conclusions:**

The CONUT and AL indices demonstrated positive associations with HF in the general population. Depressive status is a moderating factor that attenuates the association between CONUT and HF. Meanwhile, CONUT and AL are not effective predictors of HF risk under conditions of depressive status. Therefore, screening for depressive status in individuals with high CONUT and AL indices is important for predicting HF.

## 1. Introduction

Heart failure (HF) is a global cardiovascular disorder with a steadily increasing incidence worldwide. HF has emerged as one of the leading causes of hospitalization in the United States, with a steadily increasing societal burden [1]. According to the World Health Organization, the prevalence of HF among adults globally during 2017–2020 was approximately 2.5%. HF is a leading cause of disability and mortality, accounting for 13.4% of all sudden deaths in 2018 [2]. Although a declining trend in the relative mortality of HF was observed prior to 2012, the fatality rate since then has risen continuously [3]. Projections indicate that by 2030, the number of HF patients in the United States will exceed 8 million, with a prevalence approaching 3%, and particularly affecting the elderly population [4]. Consequently, further research is urgently needed to improve the prediction of HF.

Malnutrition is a common comorbidity associated with HF, with detrimental effects across diverse environmental contexts and regardless of the health status of patients. Malnutrition is also closely associated with the mortality and disability rates of cardiovascular diseases [5]. The controlling nutritional status (CONUT) index and allostatic load (AL) are two important tools for assessing an individual’s nutritional status and physiological system imbalance. Although they have shown some promise in predicting HF, their efficacy remains to be fully evaluated. Previous studies have reported a robust correlation between CONUT and HF [6, 7, 8], with the strength and direction of the association varying across different HF subtypes [9]. A significant positive correlation was observed between albumin concentration and the readmission rate, and hence albumin concentration may represent an independent risk factor for HF [10, 11]. In contrast, there is still only limited research on the relationship between AL and HF. A positive correlation was found between AL and HF, with age identified as a potential effect modifier [12]. Compared to CONUT, AL incorporates neuroendocrine components in addition to cardiovascular and metabolic parameters [13]. Whether this innovative inclusion enhances the predictive accuracy for HF warrants further investigation.

Comorbidity research in HF has recently emerged as a prominent focus in both domestic and international studies, contributing to a deeper understanding of HF pathophysiology. With regard to psychosocial factors, depression poses a significant global health burden and is characterized by persistent low mood, anhedonia, and diminished interest [14, 15]. Metabolic syndrome is a pathological condition closely associated with cardiovascular diseases. It is primarily defined by insulin resistance, atherosclerosis-related dyslipidemia, central obesity, and hypertension [16]. Recent studies have demonstrated significant correlations between components of metabolic syndrome and depression [17], with oxidative stress and inflammatory mediation playing pivotal roles [18].

In the present study, we therefore investigated whether psychosocial factors can influence the development of HF by altering metabolic profiles. We conducted subgroup analyses to examine the relationships between CONUT, AL, and HF across different depressive states using various models. These models incorporated covariates presumed to be associated with HF, including demographic and cardiovascular variables. Furthermore, by employing net reclassification improvement (NRI) and integrated discrimination improvement (IDI) methodologies, we compared the performance of the models for predicting HF, with the aim of providing clinicians with enhanced tools for HF risk stratification.

## 2. Materials and Methods

### 2.1 Study Population

**Fig. 1.**
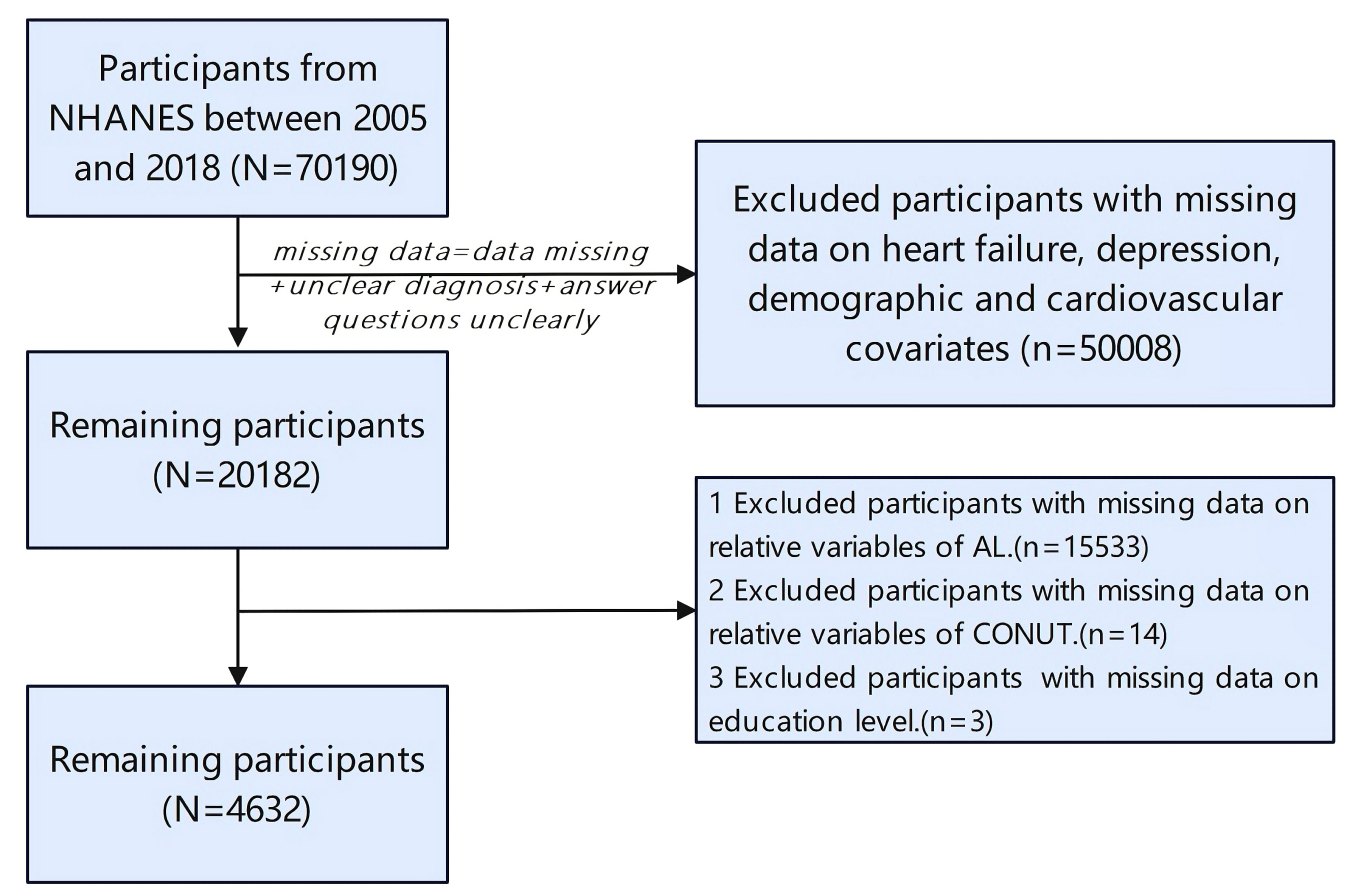
**Flowchart for the screening of study subjects**. NHANES, National Health and Nutrition Examination Survey; AL, allostatic load; CONUT, controlling nutritional status.

We analyzed data from the National Health and Nutrition Examination Survey (NHANES) (2005–2018), encompassing demographic, physical examination, laboratory testing, and questionnaire-based information. Specifically, the demographic variables included age, sex, race, income, and educational level, while the cardiovascular-related parameters included hypertension, diabetes, coronary heart disease, heart attack, anemia, and stroke. CONUT- and AL-related components were also collected. To ensure an adequate sample size, data from adults aged ≥20 years across the 14-year study period were included. For non-dichotomous variables, only cases with missing data were excluded. For dichotomous variables, cases with unclear diagnoses or missing data were excluded. Following the weighting procedure, samples that underwent no weighting adjustment were excluded from the analysis (missing data was cleaned using Excel). To ensure the inclusion of consistent variable categories across different survey years—including demographic, examination, laboratory, and questionnaire data—any change in measurement equipment (e.g., for blood pressure) was accompanied by the maintenance of uniform measurement protocols. Specifically, the anatomical measurement site, environmental conditions, and personnel training were kept standardized across all years, thereby minimizing potential biases and ensuring comparability and reliability of the recorded data. A total of 4632 participants were included in the final stage. The exclusion rate was approximately 93.4%. The detailed screening flowchart is shown in Fig. 1.

### 2.2 Outcome Variable

In the NHANES database, HF data were derived from personal interviews conducted during the questionnaire component, and specifically based on the response to question MCQ160b: “Ever told you had congestive heart failure?” A positive response (“Yes”) was coded as a confirmed HF diagnosis.

### 2.3 Exposure Variable

The CONUT score is comprised of three components: serum albumin (LBXSAL), total lymphocyte count (LBDLYMNO), and total cholesterol (LBXTC) [19]. All blood tests were performed under fasting conditions to ensure glucose measurements were not significantly deviated from actual values. The calculation formula was defined as: CONUT score = serum albumin score (g/dL) + total lymphocyte score (cells/mm^3^) + total cholesterol score (mg/dL). Based on the CONUT score, malnutrition severity was classified into four grades: normal, mild, moderate, and severe [20].

The AL score was derived from the thresholds for total cholesterol (mg/dL), high-density lipoprotein (HDL) (mg/dL), low-density lipoprotein (LDL) (mg/dL), triglycerides (mg/dL), systolic blood pressure (mmHg), diastolic blood pressure (mmHg), waist circumference (cm), body mass index (BMI) (kg/m^2^), fasting blood glucose (mg/dL), insulin (µU/mL), and serum creatinine (mg/dL). For each parameter, thresholds were defined as the first quartile (Q1) or third quartile (Q3). HDL levels below Q1 were scored as 1, while other parameters that exceeded Q3 were scored as 1. Individual scores were categorized as low AL if the sum was <3, or high AL if the sum was ≥3 [21].

Depressive status was assessed using the Patient Health Questionnaire-9 (PHQ-9), with symptoms rated on a 4-point Likert scale ranging from 0 (“not at all”) to 3 (“nearly every day”). A total score of ≥10 indicated a diagnosis of depression [22, 23].

### 2.4 Covariates

Potentially relevant covariates were selected to construct the predictive model, including age (years), sex (male/female), race (Mexican American/other Hispanic/non-Hispanic White/non-Hispanic Black/other race), education level (less than high school/high school graduate/high school graduate or above), poverty income ratio (PIR) (<5/≥5) [24], history of alcohol use, smoking history, as well as medical history of hypertension, coronary heart disease, diabetes, anemia, heart attack, and stroke. A PIR threshold of 5 serves as a practical cutoff to distinguish between individuals or households that are relatively economically vulnerable (PIR <5) and those with relatively secure economic conditions (PIR ≥5). This facilitates analysis of the impact of socioeconomic status on health outcomes, healthcare utilization, and disease risk. Drinking was defined as having consumed alcohol on at least 12 occasions during the past year, while smoking status was defined as having smoked at least 100 cigarettes over one’s lifetime.

### 2.5 Statistical Analysis

This study utilized R Studio (version 4.5.0, Posit Software, PBC, Boston, MA, USA) and SPSS (version 27.0, IBM Corporation, Armonk, NY, USA) for statistical analysis, incorporating the complex survey design and weighting procedures of NHANES data. The analysis was conducted using the participant subgroup with the smallest sample size, corresponding to the interview-weighted data (WTINT2YR). Specifically, the applied weight was calculated as one-seventh of the original interview weight, i.e., 1/7 × WTINT2YR. The stratification (strata) and clustering (primary sampling unit, PSU) variables were defined as follows: SDMVPSU (Sampling Design PSU) as the id, SDMVSTRA (Sampling Design Stratum) as the strata, and SDMVPSU as the PSU. Baseline data were stratified by HF status (yes/no), CONUT category (normal/mild/moderate), and AL level (low/high). Baseline characteristics were compared between two groups using the Mann-Whitney U test or *t* test, and the ANOVA test or Kruskal–Wallis test for comparisons across ≥3 groups. Normally distributed continuous variables were expressed as the mean ± standard error, while skewed continuous variables were expressed as the median and interquartile ranges. The determination of normality was based on whether the skewness and its standard error, as well as the kurtosis and its standard error, fall within the range of ±1.96.

Categorical variables were reported as a frequency (percentage). Statistical significance was set at *p* < 0.05.

Binary logistic regression was employed to analyze the associations between the two indices and HF. Model 1 was unadjusted; model 2 was adjusted for demographic factors (age, sex, race, education level, PIR, alcohol use history, and smoking history); model 3 was adjusted for cardiovascular risk factors (hypertension, coronary heart disease, diabetes, anemia, heart attack, and stroke); and model 4 was adjusted for both demographic and cardiovascular factors (**Supplementary Table 1**). The results are presented as odds ratios (ORs) with 95% confidence intervals (95% CI).

Subgroup analyses were conducted to explore potential effect modifiers (*p* for interaction < 0.05) influencing the associations between the two indices and heart failure. Restricted cubic splines (RCS) were employed to assess potential linear or nonlinear relationships between the indices and HF. Additionally, a multicollinearity test was performed to assess correlations among variables (e.g., ensure variance inflation factor [VIF] <5). Model performance was evaluated through receiver operating characteristic (ROC) curves, with calculation of the area under the curve (AUC), NRI, and IDI. Internal validation (bootstrapping) was performed to assess overfitting, thereby evaluating the stability of the model. Statistically significant differences in model performance for NRI and IDI comparisons were considered as *p* < 0.05.

## 3. Results

### 3.1 Baseline Characteristics

For intergroup comparisons, the baseline data were first stratified by HF status, CONUT categories, and AL levels.

Among the 4632 participants, 144 (3.1%) were diagnosed with HF (Table 1). The HF group had a significantly higher mean age compared to the non-HF group, as well as higher proportions of non-Hispanic Whites and non-Hispanic Blacks. Moreover, the HF group had significantly lower educational levels and PIR. The prevalence of cardiovascular diseases was significantly higher in the HF group. The CONUT and AL components consistently indicated poorer nutritional status and worse physiological conditions in the HF group. The prevalence of depression was significantly higher in the HF group (*p* = 0.002).

**Table 1.** **Baseline characteristics categorized according to the presence or absence of HF**.

Variables		Overall	Non-heart failure	Heart failure	*p*-value
		N = 4632 (100.0%)	N = 4488 (96.9%)	N = 144 (3.1%)	
**Age (median [IQR])**		46 (32, 62)	46 (31, 61)	68 (59, 78)	<0.001
**Sex (%)**					0.285
	Male	2050 (44.3%)	1980 (44.1%)	70 (48.6%)	
	Female	2582 (55.7%)	2508 (55.9%)	74 (51.4%)	
**Race (%)**					0.009
	Mexican American	697 (15.0%)	688 (15.3%)	9 (6.2%)	
	Other Hispanic	408 (8.8%)	404 (9.0%)	4 (2.8%)	
	Non-Hispanic White	2337 (50.5%)	2246 (50.0%)	91 (63.2%)	
	Non-Hispanic Black	904 (19.5%)	868 (19.3%)	36 (25.0%)	
	Other race-including multi-racial	286 (6.2%)	282 (6.4%)	4 (2.8%)	
**Education (%)**					0.010
	Less than high school	1199 (25.9%)	1148 (25.6%)	51 (35.4%)	
	High school	1103 (23.8%)	1070 (23.8%)	33 (22.9%)	
	More than high school	2330 (50.3%)	2270 (50.6%)	60 (41.7%)	
**PIR (%)**					<0.001
	Low income	3880 (83.8%)	3743 (83.4%)	137 (95.1%)	
	High income	752 (16.2%)	745 (16.6%)	7 (4.9%)	
**Smoking (%)**		2197 (47.4%)	2121 (47.3%)	76 (52.8%)	0.192
**Hypertension (%)**		1629 (35.2%)	1520 (33.9%)	109 (75.7%)	<0.001
**Coronary heart disease (%)**		176 (3.8%)	127 (2.8%)	49 (34.0%)	<0.001
**Diabetes (%)**		550 (11.9%)	493 (11.0%)	57 (39.6%)	<0.001
**Anemia (%)**		239 (5.2%)	217 (4.8%)	22 (15.3%)	<0.001
**Heart attack (%)**		189 (4.1%)	130 (2.9%)	59 (41.0%)	<0.001
**Stroke (%)**		193 (4.2%)	162 (3.6%)	31 (21.5%)	<0.001
**Albumin (median [IQR])**		4.2 (4.0, 4.4)	4.2 (4.0, 4.4)	4.1 (3.8, 4.3)	<0.001
**Lymphocyte (median [IQR]**)		1.9 (1.6, 2.4)	1.9 (1.6, 2.4)	1.8 (1.4, 2.3)	0.001
**Total cholesterol (median [IQR]**)		192 (165, 219)	192 (165, 219)	186 (153, 212)	0.010
**Total cholesterol-factor (%)**					0.007
	Total cholesterol ≤219 mg/dL	3487 (75.3%)	3374 (75.2%)	113 (78.5%)	
	Total cholesterol >219 mg/dL	1145 (24.7%)	1114 (24.8%)	31 (21.5%)	
**CONUT (median [IQR])**		1 (0, 1)	1 (0, 1)	1 (0, 2)	<0.001
**CONUT-factor (%)**					<0.001
	Normal	3658 (78.9%)	3567 (79.5%)	91 (63.2%)	
	Mild	946 (20.4%)	897 (20.0%)	49 (34.0%)	
	Moderate	28 (0.7%)	24 (0.5%)	4 (2.8%)	
**LDL-factor (%)**					0.192
	LDL ≤136 mg/dL	3485 (75.2%)	3370 (75.1%)	115 (79.9%)	
	LDL >136 mg/dL	1147 (24.8%)	1118 (24.9%)	29 (20.1%)	
**HDL-factor (%)**					0.053
	HDL <43 mg/dL	1102 (23.8%)	1058 (23.6%)	44 (30.6%)	
	HDL ≥43 mg/dL	3530 (76.2%)	3430 (76.4%)	100 (69.4%)	
**Triglycerides-factor (%)**					0.010
	Triglycerides ≤158 mg/dL	3481 (75.2%)	3386 (75.4%)	95 (66.0%)	
	Triglycerides >158 mg/dL	1151 (24.8%)	1102 (24.6%)	49 (34.0%)	
**FBG-factor (%)**					<0.001
	FBG ≤108 mg/dL	3475 (75.0%)	3396 (75.7%)	79 (54.9%)	
	FBG >108 mg/dL	1157 (25.0%)	1092 (24.3%)	65 (45.1%)	
**Insulin-factor (%)**					0.011
	Insulin ≤16.29 µU/mL	3475 (75.0%)	3380 (75.3%)	95 (66.0%)	
	Insulin >16.29 µU/mL	1157 (25.0%)	1108 (24.7%)	49 (34.0%)	
**Systolic blood pressure-factor (%)**					<0.001
	Systolic blood pressure ≤132 mmHg	3522 (76.0%)	3434 (76.5%)	88 (61.1%)	
	Systolic blood pressure >132 mmHg	1110 (24.0%)	1054 (23.5%)	56 (38.9%)	
**Diastolic blood pressure-factor (%)**					0.138
	Diastolic blood pressure ≤76 mmHg	3488 (75.3%)	3372 (75.1%)	116 (80.6%)	
	Diastolic blood pressure >76 mmHg	1144 (24.7%)	1116 (24.9%)	28 (19.4%)	
**Waist circumference-factor (%)**					<0.001
	Waist circumference ≤108.8 cm	3479 (75.1%)	3395 (75.6%)	84 (58.3%)	
	Waist circumference >108.8 cm	1153 (24.9%)	1093 (24.4%)	60 (41.7%)	
**BMI-factor (%)**					0.537
	BMI ≤32.6 kg/m^2^	3479 (75.1%)	3374 (75.2%)	105 (72.9%)	
	BMI >32.6 kg/m^2^	1153 (24.9%)	1114 (24.8%)	39 (27.1%)	
**Serum creatinine-factor (%)**					<0.001
	Serum creatinine ≤1 mg/dL	3647 (78.7%)	3579 (79.7%)	68 (47.2%)	
	Serum creatinine >1 mg/dL	1153 (21.3%)	909 (20.3%)	76 (52.8%)	
**AL (median [IQR])**		2 (1, 4)	2 (1, 4)	4 (2, 5)	<0.001
**AL-factor (%)**					<0.001
	Low AL	2392 (51.6%)	2346 (52.3%)	46 (31.9%)	
	High AL	2240 (48.4%)	2142 (47.7%)	98 (68.1%)	
**Depression score (median [IQR])**		3 (2, 6)	3 (2, 6)	5 (2, 9)	<0.001
**Depression score-factor (%)**					0.002
	Yes score ≥10	630 (13.6%)	598 (13.3%)	32 (22.2%)	
	No score <10	4002 (86.4%)	3890 (86.7%)	112 (77.8%)	

HF, heart failure; PIR, poverty income ratio; CONUT, controlling nutritional status; LDL, low-density lipoprotein; HDL, high-density lipoprotein; FBG, fasting blood glucose; BMI, body mass index; AL, allostatic load; IQR, interquartile range.

Next, participants were stratified according to CONUT categories, with 3658 in the normal group, 946 in the mild malnutrition group, and 28 in the moderate malnutrition group (**Supplementary Table 2**). Significant racial disparities were observed across groups, with the moderate malnutrition group having a higher proportion of non-Hispanic Blacks. The moderate malnutrition group also exhibited significantly higher prevalence rates for other cardiovascular diseases and conditions compared to the other CONUT groups, with the exception of diabetes and stroke. The prevalence of HF increased progressively with the aggravation of malnutrition severity (*p* < 0.001).

When stratified by AL levels, 2392 participants were in the low AL group and 2240 in the high AL group (**Supplementary Table 3**). Compared to the low AL group, the high AL group was significantly older, had a higher proportion of males, had lower education levels and PIR, and a higher prevalence of smoking. The high AL group also showed a significantly higher prevalence of cardiovascular diseases and HF, as well as a higher prevalence of depression (*p* = 0.002).

### 3.2 Associations Between CONUT, AL, and HF

Binary logistic regression was performed to analyze the associations between CONUT/AL and HF after adjusting for demographic or cardiovascular risk factors.

**Fig. 2.**
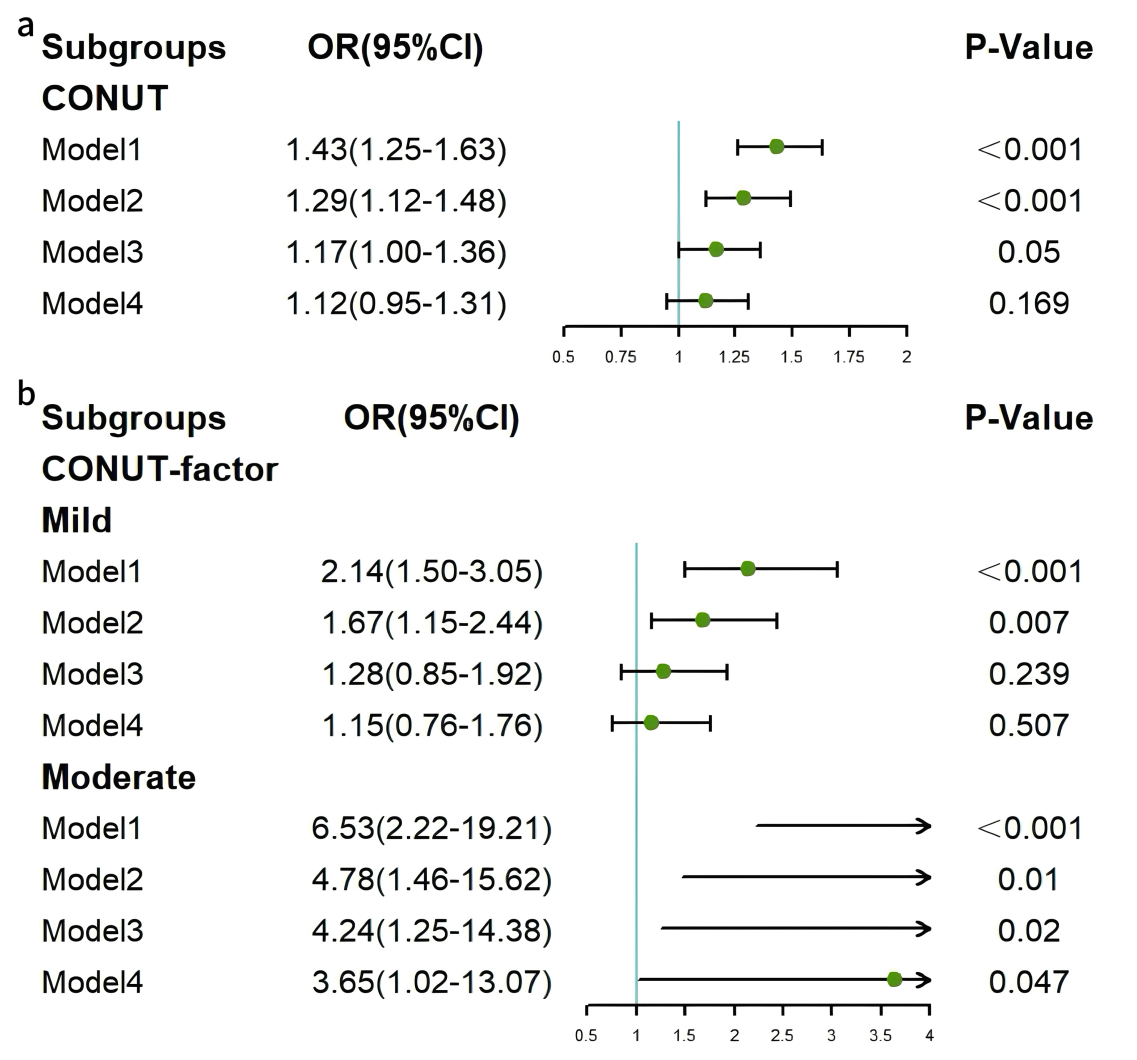
**Forest plots of associations between CONUT and HF**. (a) Continuous CONUT and HF. (b) Categorical CONUT and HF. HF, heart failure; CONUT, controlling nutritional status.

To investigate the association between continuous CONUT and HF, binary logistic regression analyses were conducted across four progressively adjusted models. Model 1 (unadjusted) showed a significant positive association between CONUT and HF (OR = 1.43, 95% CI: 1.25–1.63, *p* < 0.001). Model 2 (adjusted for demographic factors) also demonstrated a consistent positive association (OR = 1.29, 95% CI: 1.12–1.48, *p* < 0.001). Models 3 and 4 revealed no significant association between CONUT and HF (*p* > 0.05). Stratified analyses were subsequently conducted according to the CONUT category, with the normal group as the reference. In the mild malnutrition group, significant associations were observed in Model 1 (OR = 2.14, 95% CI: 1.50–3.05, *p* < 0.001) and Model 2 (OR = 1.67, 95% CI: 1.15–2.44, *p* = 0.007), but not in Models 3 and 4. In the moderate malnutrition group, OR values in all models were significantly higher than those in the mild malnutrition group, with persistent significant associations between CONUT and HF (*p* < 0.05) (Fig. 2).

**Fig. 3.**
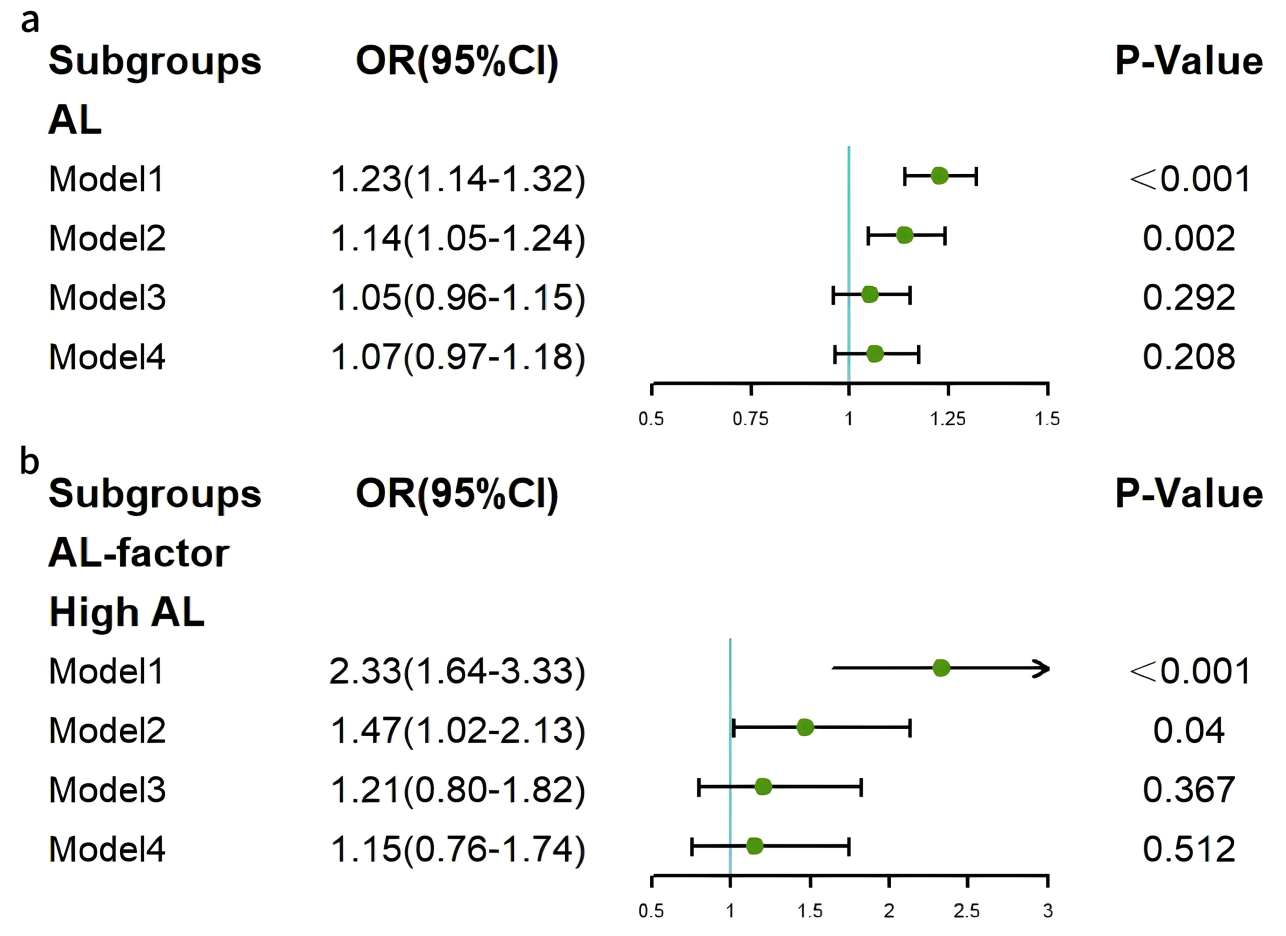
**Forest plots of associations between AL and HF**. (a) Continuous AL and HF. (b) Categorical AL and HF. HF, heart failure; AL, allostatic load.

Binary logistic regression analyses were also conducted to investigate the association between continuous AL and HF. This revealed significant positive associations in Model 1 (OR = 1.23, 95% CI: 1.14–1.32, *p* < 0.001) and Model 2 (OR = 1.14, 95% CI: 1.05–1.24, *p* = 0.002), but not in Models 3 and 4. Stratified analyses according to AL levels were also performed, with the low AL group as the reference. In the high AL group, significant associations were observed in Model 1 (OR = 2.33, 95% CI: 1.64–3.33, *p* < 0.001) and Model 2 (OR = 1.47, 95% CI: 1.02–2.13, *p* = 0.04), but not in Models 3 and 4 (*p* > 0.05) (Fig. 3).

### 3.3 Analysis of Subgroups With Different Depression Status

**Fig. 4.**
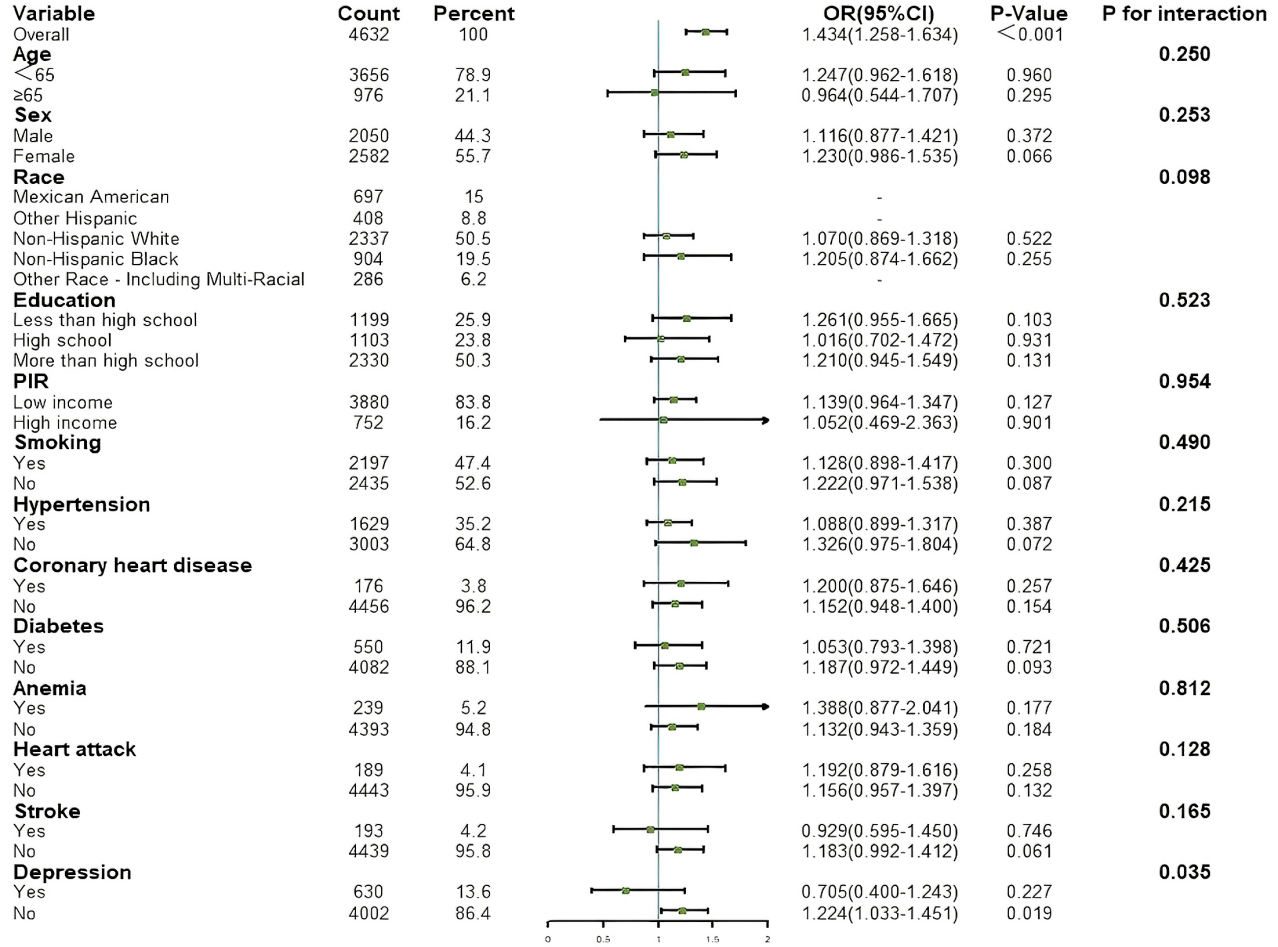
**Results of subgroup analysis for associations between CONUT and HF**. HF, heart failure; CONUT, controlling nutritional status; PIR, poverty income ratio.

**Fig. 5.**
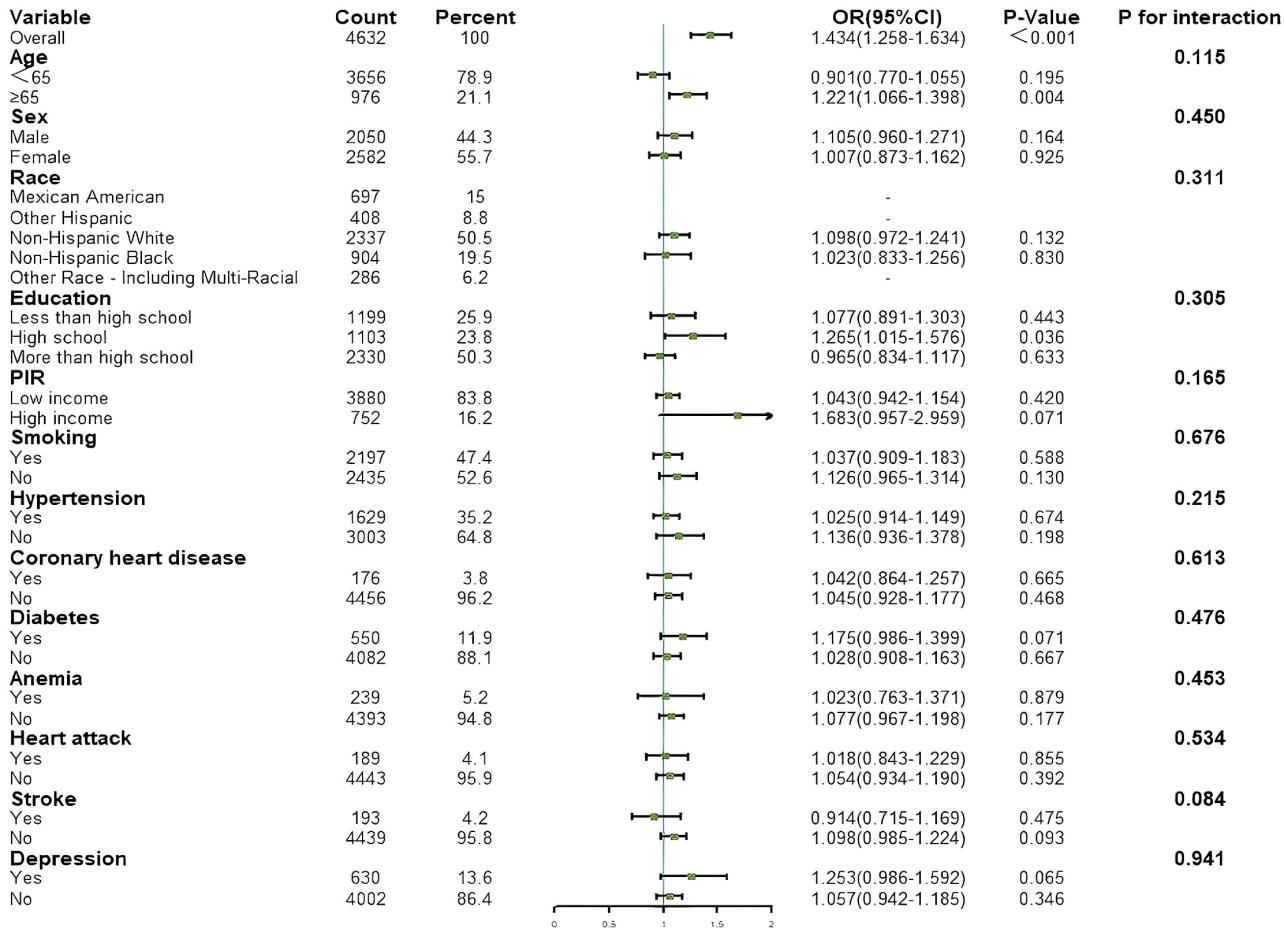
**Results of subgroup analysis for associations between AL and HF**. AL, allostatic load; HF, heart failure; PIR, poverty income ratio.

Subgroup analyses were conducted to explore potential effect modifiers that could influence the association between the two indices and HF (results shown in Figs. 4,5). Depression was identified as a significant effect modifier in the relationship between CONUT and HF (*p* for interaction = 0.035). In the non-depression subgroup, CONUT was significantly associated with HF (OR = 1.224, 95% CI: 1.033–1.451, *p* = 0.019). In the subgroup of participants aged ≥65 years, AL was significantly associated with HF (OR = 1.221, 95% CI: 1.066–1.398, *p* = 0.004). Stratified analyses by depressive status were also conducted to examine the associations between CONUT/AL and HF across different depression subgroups. The results for each model are shown in Tables 2,3.

**Table 2.** **Baseline characteristics categorized by CONUT levels**.

		Subgroup	OR (95% CI)	*p*-value
**CONUT**		**Depression**		
	Model 1	No	1.558 (1.354–1.792)	<0.001
		Yes	0.950 (0.645–1.400)	0.797
	Model 2	No	1.389 (1.195–1.615)	<0.001
		Yes	0.909 (0.594–1.392)	0.661
	Model 3	No	1.255 (1.065–1.479)	0.007
		Yes	0.869 (0.548–1.379)	0.550
	Model 4	No	1.224 (1.033–1.451)	0.019
		Yes	0.705 (0.400–1.243)	0.227
**CONUT-factor**		**Depression**		
**Mild**				
	Model 1	No	2.633 (1.777–3.901)	<0.001
		Yes	0.913 (0.368–2.269)	0.845
	Model 2	No	2.046 (1.349–3.104)	<0.001
		Yes	0.819 (0.309–2.174)	0.689
	Model 3	No	1.522 (0.972–2.382)	0.066
		Yes	0.789 (0.271–2.295)	0.663
	Model 4	No	1.459 (0.918–2.317)	0.110
		Yes	0.540 (0.152–1.920)	0.341
**Moderate**				
	Model 1	No	9.061 (3.025–27.141)	<0.001
		Yes	-	-
	Model 2	No	6.546 (1.946–22.017)	0.002
		Yes	-	-
	Model 3	No	5.273 (1.530–18.172)	0.008
		Yes	-	-
	Model 4	No	4.710 (1.302–17.041)	0.018
		Yes	-	-

CONUT, controlling nutritional status; OR, odds ratio; CI, confidence interval.

**Table 3.** **Baseline characteristics categorized by AL level**.

		Subgroup	OR (95% CI)	*p*-value
**AL**		**Depression**		
	Model 1	No	1.235 (1.138–1.341)	<0.001
		Yes	1.161 (0.998–1.350)	0.053
	Model 2	No	1.140 (1.034–1.256)	0.009
		Yes	1.129 (0.948–1.345)	0.174
	Model 3	No	1.051 (0.945–1.170)	0.356
		Yes	1.071 (0.885–1.297)	0.482
	Model 4	No	1.063 (0.943–1.199)	0.318
		Yes	1.588 (0.977–2.582)	0.062
**AL-factor**		**Depression**		
**High AL**				
	Model 1	No	2.394 (1.602–3.576)	<0.001
		Yes	1.924 (0.896–4.133)	0.093
	Model 2	No	1.457 (0.959–2.213)	0.078
		Yes	1.440 (0.644–3.220)	0.375
	Model 3	No	1.190 (0.750–1.887)	0.460
		Yes	1.268 (0.509–3.159)	0.611
	Model 4	No	1.087 (0.681–1.736)	0.725
		Yes	1.505 (0.540–4.195)	0.435

AL, allostatic load; OR, odds ratio; CI, confidence interval.

In the non-depressive state, ORs for continuous CONUT were consistently >1 across all models. When CONUT was categorized into levels (with the normal group as the reference), the ORs for the mild malnutrition group remained >1 in Models 1 and 2. All models showed ORs >1 for the moderate malnutrition group. Notably, the ORs tended to increase with escalating severity of malnutrition.

In the non-depressive state, the ORs for continuous AL were >1 in Models 1 and 2. When AL was categorized into levels (with the low AL group as the reference), the OR for the high AL group was significantly elevated in Model 1. However, neither CONUT nor AL demonstrated significant associations with HF across all models in the depressive state.

### 3.4 Analysis of RCS Plots

**Fig. 6.**
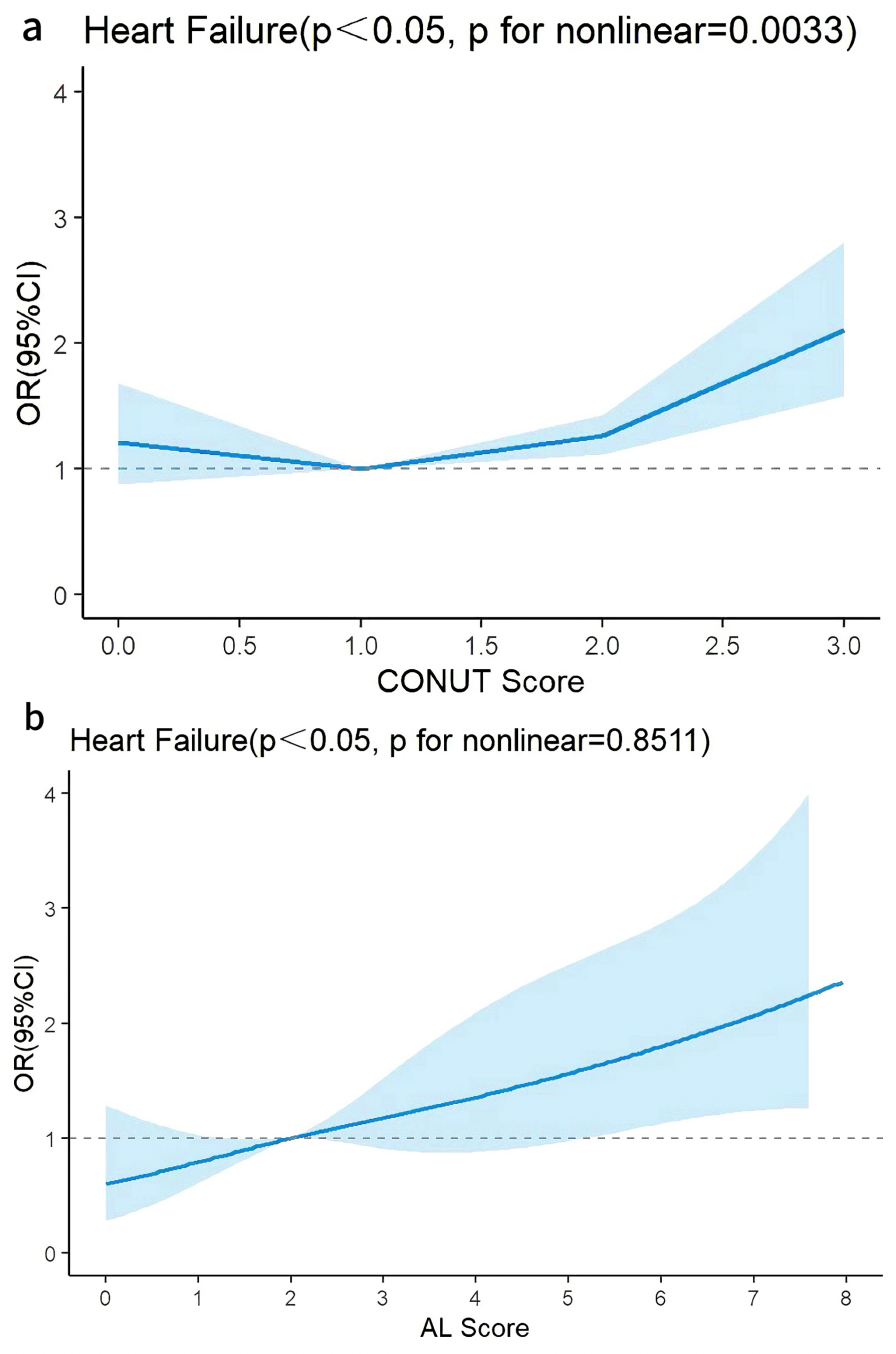
**Restricted cubic spline plots were used to assess linear or nonlinear relationships between CONUT, AL, and heart failure**. (a) RCS plot of CONUT in Model 2. (b) RCS plot of AL in Model 2 (adjusted for the demographic factors of age, sex, race, education level, poverty income ratio, alcohol use history, and smoking history). RCS, restricted cubic spline; CONUT, controlling nutritional status; AL, allostatic load.

RCS were employed to examine potential linear relationships between the two indices and HF (Fig. 6a,b). To minimize estimation bias in baseline analyses, three knot points were selected for categorical classification at quantiles of the distribution.

After adjusting for demographic variables, continuous CONUT demonstrated a nonlinear relationship with HF (*p* for overall < 0.05, *p* for nonlinear = 0.0033). Threshold effect analysis identified a critical threshold value of 1.5 (**Supplementary Table 4**).

Specifically, when CONUT was <1.5, the ORs for HF showed an inverse trend with increasing CONUT. Conversely, when CONUT reached or exceeded 1.5, the ORs for HF showed a significant positive association with elevated CONUT.

After adjusting for demographic factors, continuous AL demonstrated a linear relationship with HF (*p* for overall < 0.05, *p* for nonlinear = 0.8511). The ORs for HF exhibited a progressively increasing trend with higher AL values.

### 3.5 Comparison of Models by ROC, NRI, and IDI

**Fig. 7.**
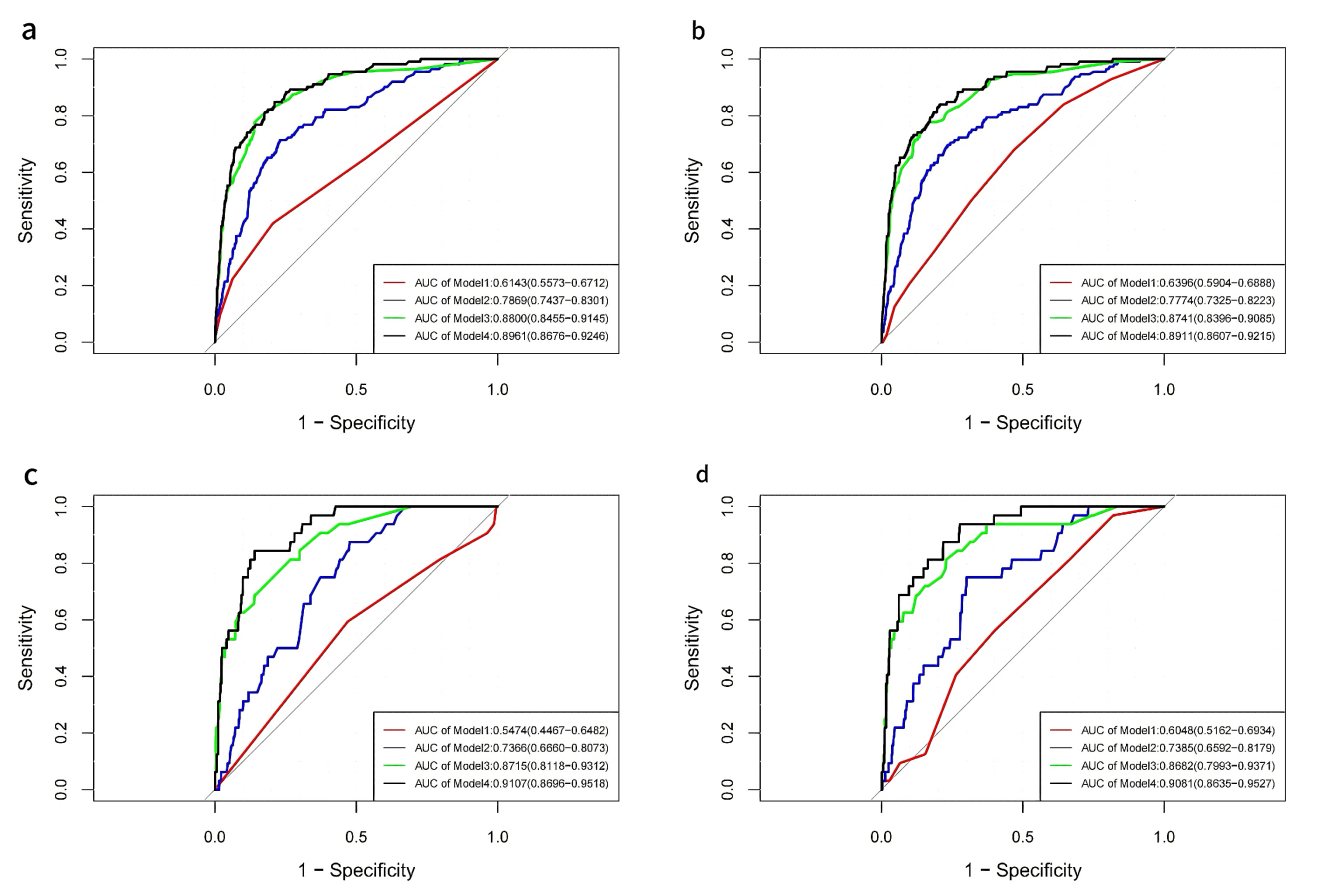
**ROC curves were used to assess the efficacy of models with CONUT and AL, and to calculate the cutoff values**. (a) ROC curves of CONUT in the four models for participants without depression. (b) ROC curves of AL in the four models for participants without depression. (c) ROC curves of CONUT in the four models for participants with depression. (d) ROC curves of AL in the four models for participants with depression. ROC, receiver operating characteristic; AUC, area under the curve; CONUT, controlling nutritional status; AL, allostatic load.

ROC curves were constructed to calculate AUC values and optimal cutoff values for both indices across all models (Fig. 7a–d). In the depressive state, significant AUC values for CONUT were observed in all models except Model 1 (where the AUC value included 0.5), while AL showed significant AUC values across all models. In the non-depressive state, CONUT and AL exhibited significant AUC values in all models, except for Model 1 where the CONUT sensitivity was <50% (Table 4). Specific cutoff values were identified as follows: for CONUT in the normal group, the cutoff was 1.500 (sensitivity 79.50%, specificity 42.00%); for AL in the normal group, the cutoff was 2.500 (sensitivity 53.10%, specificity 67.90%); and for AL in the depressive group, the cutoff was 3.500 (sensitivity 59.90%, specificity 56.20%). Multicollinearity diagnostics revealed the VIF was <5 for both CONUT and AL across all model subgroups, suggesting model robustness. AL (depression): original AUC = 0.9081, Bootstrap AUC mean = 0.8639, AUC bias (original - Bootstrap mean) = 0.0442; CONUT (depression): original AUC = 0.9107, Bootstrap AUC mean = 0.8639, AUC bias (original - Bootstrap mean) = 0.0468; AL (non-depression): original AUC = 0.8910, Bootstrap AUC mean = 0.8814, AUC bias (original - Bootstrap mean) = 0.00968; CONUT (non-depression): original AUC = 0.8961, Bootstrap AUC mean = 0.8861, AUC bias (original - Bootstrap mean) = 0.0100. As the AUC values for CONUT and AL were similar in Model 4, NRI and IDI were applied to compare their predictive performance for HF. No significant difference in predictive performance was found between the two indices in Model 4 after adjusting for all covariates (*p* > 0.05) (Table 5).

**Table 4.** **Comparison of CONUT and AL efficacy for the association with HF (ROC curves)**.

		ROC (CONUT Score)			ROC (AL Score)		
		AUC [95% CI]	Sensitivity	Specificity	AUC [95% CI]	Sensitivity	Specificity
**Subgroup 1: Depression**							
	Model 1	0.5474 (0.4467–0.6482)	59.40%	53.00%	0.6048 (0.5162–0.6934)	56.20%	59.90%
	Model 2	0.7366 (0.6660–0.8073)	87.50%	52.30%	0.7385 (0.6592–0.8179)	75.00%	69.90%
	Model 3	0.8715 (0.8118–0.9312)	68.80%	86.00%	0.8682 (0.7993–0.9371)	81.20%	77.10%
	Model 4	0.9107 (0.8696–0.9518)	84.40%	86.00%	0.9081 (0.8635–0.9527)	93.80%	72.20%
**Subgroup 2: Non-Depression**							
	Model 1	0.6143 (0.5573–0.6712)	42.00%	79.50%	0.6396 (0.5904–0.6888)	67.90%	53.10%
	Model 2	0.7869 (0.7437–0.8301)	71.40%	77.00%	0.7774 (0.7325–0.8223)	69.60%	76.70%
	Model 3	0.8800 (0.8455–0.9145)	77.70%	86.00%	0.8741 (0.8396–0.9085)	76.80%	84.10%
	Model 4	0.8961 (0.8676–0.9246)	84.80%	78.90%	0.8911 (0.8607–0.9215)	83.90%	79.50%

CONUT, controlling nutritional status; AL, allostatic load; ROC, receiver operating characteristic; AUC, area under the curve; CI, confidence interval; HF, heart failure.

**Table 5.** **Comparison of CONUT and AL efficacy for the association with HF (NRI/IDI)**.

		NRI		IDI	
		NRI [95% CI]	*p*-value	IDI [95% CI]	*p*-value
**Subgroup 1: Depression**					
	Model 1	–0.0179 (–0.1552–0.0443)	0.7255	–0.0062 (–0.0131–0.0007)	0.0803
	Model 2	–0.0625 (–0.1712–0.1116)	0.3719	–0.0055 (–0.0153–0.0043)	0.2713
	Model 3	–0.0268 (–0.1334–0.0614)	0.5845	–0.0022 (–0.0099–0.0055)	0.5720
	Model 4	–0.0982 (–0.1267–0.0794)	0.0585	–0.0025 (–0.0105–0.0054)	0.5308
**Subgroup 2: Non-Depression**					
	Model 1	–0.0313 (–0.1476–0.4535)	0.8489	0.0056 (–0.0009–0.0121)	0.0913
	Model 2	0.1563 (–0.1481–0.4000)	0.2632	0.0095 (–0.0012–0.0201)	0.0810
	Model 3	0.0625 (–0.0953–0.3030)	0.5282	–0.0037 (–0.0136–0.0063)	0.4714
	Model 4	0.0625 (–0.1713–0.2601)	0.5610	–0.003 (–0.0217–0.0158)	0.7569

NRI, net reclassification improvement; IDI, integrated discrimination improvement; CI, confidence interval; CONUT, controlling nutritional status; AL, allostatic load; HF, heart failure.

## 4. Discussion

HF is a clinical syndrome characterized by impaired capacity for cardiac pumping function or filling. This disease imposes a substantial socioeconomic burden in the United States and globally, with rising annual incidence and mortality rates [15]. The 2021 American Heart Association Heart Disease and Stroke Statistics Update projected that the prevalence of HF in the U.S. was likely to increase from 2.4% in 2012 to 3% in 2030, while the total treatment costs would increase from 30.7 billion dollars in 2012 to 69.8 billion dollars in 2030 [4, 25]. HF is therefore a critical public health challenge demanding urgent attention. To better understand the risk factors associated with HF, the current study incorporated two nutritional and physiological assessment indices—CONUT and AL—while taking into account the potential influence of psychiatric factors. Specifically, depression was evaluated as a possible mediating variable due to its potential effect on metabolic components.

The CONUT and AL indices demonstrated significant positive associations with HF in this study. Compared to Model 1 (unadjusted) the ORs for both indices showed a downward trend across models that were adjusted for demographic and cardiovascular factors, suggesting potential confounding effects. When categorized by CONUT and AL levels, significantly elevated ORs were observed in the high CONUT/AL groups compared to their low-level counterparts. Notably, the moderate malnutrition subgroup in the CONUT stratification maintained a significant association with HF after adjusting for all confounders, consistent with this factor being closely related to HF. These findings align with similar conclusions reported by Chen *et al*. [9] and Agra Bermejo *et al*. [26].

Our subgroup analyses identified depressive status as a potential biological regulatory factor that attenuated the association between CONUT and HF. CONUT comprises serum albumin, total lymphocyte count, and total cholesterol levels, while depressive symptoms show positive correlations with metabolic abnormalities such as reduced HDL, increased lymphocyte count, and hyperlipidemia [17, 24, 27]. Although the serum albumin concentration shows a negative correlation with the incidence of depression [28], the low prevalence of hypoalbuminemia in the sample population contributed to an overall downward trend in CONUT associated with depression in the NHANES database cohort. Individuals with depression exhibit irregular eating patterns, leading to nutritional imbalances characterized by excessive intake of high-calorie, high-fat, and low-protein foods [29]. This is likely to contribute to a decline in CONUT. Currently, there is both domestic and international consensus on the positive correlation between depression and HF. Individuals with depression can activate the hypothalamic-pituitary-adrenal (HPA) axis and the sympathetic-adrenal medullary (SAM) axis. This directly induces myocardial cell hypertrophy and fibrosis, as well as increasing myocardial oxygen consumption, thereby promoting myocardial remodeling and accelerating the onset and progression of HF. The renin-angiotensin-aldosterone system (RAAS) is also activated, which induces vasoconstriction, increases cardiac afterload, and accelerates myocardial remodeling. Individuals with depression maintain a chronic low-grade inflammatory state, characterized by the sustained release of pro-inflammatory cytokines such as interleukin (IL)-6, which contributes to myocardial fibrosis and impairs myocardial contractility [30]. The attenuation of the CONUT-HF association under depressive states, as a hypothetical finding, warrants further validation through large-scale longitudinal studies.

We used RCS curves to visualize the relationship between continuous CONUT/AL and HF. This revealed a nonlinear association for CONUT, with a critical threshold value of 1.5. Beyond this threshold, CONUT emerged as a potential moderating factor for HF. Below the threshold, the ORs for HF exhibited a gradual decline with increasing CONUT. The nutritional status in the population under the threshold is relatively good or only mildly inadequate, and hence the body still has the compensatory capacity to maintain myocardial structure and function. This may explain why we observed a transient downward trend. However, once the threshold is exceeded, the degree of malnutrition worsens, the body enters a state of decompensation, and myocardial structure and function can undergo accelerated degeneration. As a consequence, the ORs for association with HF showed a significant increase. Secondly, factors such as reverse causality or survival bias may be involved. In cases of mild malnutrition, the body remains in a compensated state. This could lead to a transient increase in nutritional intake, explaining why the ORs exhibited a gradual decline before reaching the threshold. Finally, after exceeding the threshold, the nutritional status declined sharply, leading to poorer patient prognosis, increased short-term mortality, and study withdrawal, thereby indirectly introducing selection bias in the sample. Although attenuated at lower levels, the overall OR trend for HF progressively increased, with a steeper increase post-threshold compared to the pre-threshold decline, while maintaining OR values >1. These findings suggest that severe malnutrition significantly increases the risk of HF, which calls for increased monitoring of individuals with elevated CONUT to reduce mortality. This conclusion agrees with those of earlier studies that employed nutritional indices for HF prediction [6, 31, 32]. However, the potential HF-protective effect of low CONUT requires validation in prospective cohort studies. In contrast, AL exhibited a linear relationship with HF, showing a progressive increase in the OR with rising AL values. The high AL group demonstrated significantly stronger associations with HF than the low AL group. As an indicator of cumulative physiological stress burden, AL has previously been linked to both preserved and reduced ejection fraction HF [12]. The current study integrated demographic and cardiovascular factors into the analysis, building upon prior experience that combined physiological and socioenvironmental parameters [4, 33, 34, 35]. Although we observed a decrease in the OR for the association of AL with HF after demographic adjustment, the specificity and sensitivity of this predictive model were substantially improved.

Our study compared the predictive efficacy of CONUT and AL for HF using ROC curves, AUC, NRI, and IDI. In the depressive population, AL-based prediction models demonstrated superior performance over CONUT in both the unadjusted model and in models adjusted for demographic factors. The predictive performance of CONUT models improved significantly following adjustment for cardiovascular risk factors, surpassing that of AL models adjusted for comparable factors. Among non-depressed individuals, the unadjusted AL model initially outperformed CONUT. However, following adjustments for demographic and cardiovascular factors, the predictive performance of CONUT models showed significant improvement and exceeded that of AL models. Notably, when demographic and cardiovascular factors were jointly adjusted, the AUC values of AL and CONUT reached optimally comparable levels. The inclusion of additional factors may capture more predictive information, thereby enhancing the model’s discriminative ability. In the non-depression subgroup, the predictive performance after incorporating additional factors was generally higher in the CONUT-related models compared to the AL-related models. However, the opposite pattern was observed in the depression subgroup. Subgroup analysis exploring the relationships between CONUT, AL, and HF did not identify any specific mediating factors, except that depression status was found to modulate the association between CONUT and HF. Further comparison of the models with NRI and IDI revealed no significant differences between the two models in terms of their efficacy of HF prediction across different depressive states. These findings suggest that adjusting for demographic and cardiovascular factors substantially improves the predictive performance of both CONUT and AL models. The integration of nutritional-metabolic and physiological homeostasis indicators, supplemented by socioenvironmental and psychiatric factors, offers good perspectives for the prediction of HF.

One of the strengths of this study was the inclusion of depressive status as a mediating factor in determining the predictive efficacy of CONUT and AL for HF. We propose a novel framework that incorporates psychiatric factors into HF prediction, thereby advancing the interdisciplinary integration of mental health and cardiovascular disease research. While CONUT serves as a risk factor for HF, our proposed CONUT prediction model also takes into account the influence of depression. Therefore, researchers should actively stratify samples according to depression status in order to reduce the occurrence of false-negative results.

This study had several limitations. Firstly, the small HF sample sizes in the high CONUT and AL groups could introduce bias. Further research with larger cohorts is required to ensure adequate statistical power. Secondly, the cross-sectional study design prevented the investigation of causal relationships between the indices and HF, highlighting the need for longitudinal designs that incorporate temporal dynamics. Thirdly, the cross-sectional design invariably led to the inclusion of confounding factors that may affect the associations between CONUT/AL and HF, thereby reducing the precision and sensitivity of our predictive models. Fourth, the NHANES database does not include specific clinical diagnostic data for HF or N-terminal pro–B-type natriuretic peptide (NT-proBNP) results related to HF. Consequently, it was not possible to assess diagnostic consistency for HF using metrics such as kappa ≈ 0.7, or to conduct sensitivity analyses. Fifth, the relatively small sample size of the moderately to severely malnourished population may limit the robustness of the conclusions. Finally, the diagnosis of depression in this study was primarily based on a PHQ ≥10, without the support of auxiliary diagnostic criteria such as clinical symptoms. This approach may introduce diagnostic bias, thereby compromising the reliability of conclusions regarding the mediating role of depressive status.

## 5. Conclusions

In the general population, CONUT and AL both emerged as potential moderating factors for HF. However, the associations between the two indices and HF differed significantly across different depressive states. While the positive correlation persisted in the non-depressed population, the association was attenuated in the depressed population.

Subgroup analyses of the included factors further confirmed that depression acts as a potential biological regulatory factor, influencing the positive association between CONUT and HF. While CONUT and AL are both clearly excellent predictors of HF, depressive status also appears to play a pivotal role. Therefore, it is essential to consider the influence of psychosocial factors on the predictive value of CONUT and AL for HF. Nutritional-metabolic, socio-environmental, and psychological factors should all be incorporated into predictive models.

## Availability of Data and Materials

The study’s original contributions are contained within the article/supplementary material. For additional information, please contact the corresponding authors.

## Supplementary Material

Supplementary material associated with this article can be found, in the online version, at https://doi.org/10.31083/RCM45879.
