# Supplementary material for: Associations Between Controlling Nutritional Status and Allostatic Load With Heart Failure Across Different Depressive States: A Cross-Sectional Study Using NHANES 2005–2018 Data
Source: Rev Cardiovasc Med. 2026 Feb 3;27(2):45879. doi: 10.31083/RCM45879 (PMC12960005; doi:10.31083/RCM45879)
Supplement: Supplementary file 1 [file 2153-8174-27-2-45879-s1.zip › Supplementary Material.docx]

**Supplementary material**

**Supplementary Table 1**

| **CONUT** | **Covariate** | **Covariate category** |
| --- | --- | --- |
| Model1 | None（0） | - |
| Model2 | age, sex, race, education level, poverty income ratio [PIR], alcohol use history, and smoking history（A） | demographic factors |
| Model3 | hypertension, coronary heart disease, diabetes, anemia, heart attack, and stroke（B） | cardiovascular factors |
| Model4 | （A）+（B） | demographic+cardiovascular factors |
| **AL** |  |  |
| Model1 | None（0） | - |
| Model2 | age, sex, race, education level, poverty income ratio [PIR], alcohol use history, and smoking history（A） | demographic factors |
| Model3 | hypertension, coronary heart disease, diabetes, anemia, heart attack, and stroke（B） | cardiovascular factors |
| Model4 | （A）+（B） | demographic+cardiovascular factors |

Age：RIDAGEYR; Sex：RIAGENDR; Race：RIDRETH1; Educational level：DMDEDUC2; Alcohol use history：ALQCOL; Smoking history：SMQ020; Hypertension：BPQ020; Coronary heart disease：MCQ160C; Diabetes：DIQ010; Anemia：MCQ053; Heart attack：MCQ160E; Stroke：MCQ160F

| **Supplementary Table 2 Baseline characteristics categorized by CONUT levels** | | | | | |
| --- | --- | --- | --- | --- | --- |
| **Variables** | **Overall N=4632 (100%)** | **CONUT -Normal N=3658(78.9%)** | **CONUT -Mild N=946(20.4%)** | **CONUT -Moderate N=28(0.7%)** | **P-Value** |
| ***Age (median [IQR])*** | 46(32，62) | 46(32，62) | 46(30，68) | 53.50(31.00,75.25) | 0.268 |
| ***Sex (%)*** |  |  |  |  | 0.354 |
| Male | 2050(44.3%) | 1613（44.1%） | 428(45.2%) | 9(32.1%) |  |
| Female | 2582(55.7%) | 2045（55.9%） | 518(54.8%) | 19(67.9%) |  |
| ***Race (%)*** |  |  |  |  | 0.036 |
| Mexican American | 697(15.0%) | 579（15.8%） | 113(11.9%) | 5(17.9%) |  |
| Other Hispanic | 408(8.8%) | 340（9.3%） | 67(7.1%) | 1(3.6%) |  |
| Non-Hispanic White | 2337(50.5%) | 1806（49.4%） | 518(54.8%) | 13(46.4%) |  |
| Non-Hispanic Black | 904(19.5%) | 704（19.2%） | 192(20.2%) | 8(28.6%) |  |
| Other Race - Including Multi-Racial | 286(6.2%) | 229（6.3%） | 56(6.0%) | 1(3.5%) |  |
| ***Education (%)*** |  |  |  |  | 0.357 |
| Less than high school | 1199(25.9%) | 962（26.3%） | 227(24.0%) | 10(35.7%) |  |
| High school | 1103(23.8%) | 862（23.6%） | 235(24.8%) | 6(21.4%) |  |
| More than high school | 2330(50.3%) | 1834（50.1%） | 484(51.2%) | 12(42.9%) |  |
| ***PIR (%)*** |  |  |  |  | 0.839 |
| Low income | 3880(83.8%) | 3070（83.9%） | 787(83.2%) | 23(82.1%) |  |
| High income | 752(16.2%) | 588（16.1%） | 159(16.8%) | 5(17.9%) |  |
| ***Smoking (%)*** | 2197(47.4%) | 1753（47.9%） | 430(45.5%) | 14(50.0%) | 0.385 |
| ***Hypertension (%)*** | 1629(35.2%) | 1242（34.0%） | 375(39.6%) | 12(42.9%) | 0.003 |
| ***Coronary heart disease (%)*** | 176(3.8%) | 100（2.7%） | 72(7.6%) | 4(14.3%) | ＜0.001 |
| ***Diabetes (%)*** | 550(11.9%) | 387（10.6%） | 159(16.8%) | 4(14.3%) | ＜0.001 |
| ***Anemia (%)*** | 239(5.2%) | 151（4.1%） | 82(8.7%) | 6(21.4%) | ＜0.001 |
| ***Heart attack (%)*** | 189(4.1%) | 119（3.3%） | 67(7.1%) | 3(10.7%) | ＜0.001 |
| ***Stroke (%)*** | 193(4.2%) | 140（3.8%） | 51(5.4%) | 2(7.1%) | 0.073 |
| ***Total cholesterol-factor (%)*** |  |  |  |  | ＜0.001 |
| Total cholesterol≤219mg/dL | 3487(75.3%) | 2619(71.6%) | 846(89.4%) | 22(78.6%) |  |
| Total cholesterol＞219mg/dL | 1145(24.7%) | 1039(28.4%) | 100(10.6%) | 6(21.4%) |  |
| ***LDL-factor (%)*** |  |  |  |  | ＜0.001 |
| LDL≤136mg/dL | 3485(75.2%) | 2595(70.9%) | 868(91.8%) | 22(78.6%) |  |
| LDL＞136mg/dL | 1147(24.8%) | 1063(29.1%) | 78(8.2%) | 6(21.4%) |  |
| ***HDL-factor (%)*** |  |  |  |  | 0.011 |
| HDL＜43mg/dL | 1102(23.8%) | 840(23.0%) | 258(27.3%) | 4(14.3%) |  |
| HDL≥43mg/dL | 3530(76.2%) | 2818(77.0%) | 688(72.7%) | 24(85.7%) |  |
| ***Triglycerides-factor (%)*** |  |  |  |  | ＜0.001 |
| Triglycerides≤158mg/dL | 3481(75.2%) | 2688(73.5%) | 773(81.7%) | 20(71.4%) |  |
| Triglycerides＞158mg/dL | 1151(24.8%) | 970(26.5%) | 173(18.3%) | 8(28.6%) |  |
| ***FBG-factor (%)*** |  |  |  |  | 0.072 |
| FBG≤108mg/dL | 3475(75.0%) | 2771(75.8%) | 685(72.4%) | 19(67.9%) |  |
| FBG＞108mg/dL | 1157(25.0%) | 887(24.2%) | 261(27.6%) | 9(32.1%) |  |
| ***Insulin-factor (%)*** |  |  |  |  | 0.124 |
| Insulin≤16.29μU/mL | 3475(75.0%) | 2735(74.8%) | 723(76.4%) | 17(60.7%) |  |
| Insulin＞16.29μU/mL | 1157(25.0%) | 923(25.2%) | 223(23.6%) | 11(39.3%) |  |
| ***Systolic blood pressure-factor (%)*** |  |  |  |  | 0.99 |
| Systolic blood pressure≤132mmHg | 3522(76.0%) | 2781(76.0%) | 720(76.1%) | 21(75.0%) |  |
| Systolic blood pressure＞132mmHg | 1110(24.0%) | 877(24.0%) | 226(23.9%) | 7(25.0%) |  |
| ***Diastolic blood pressure-factor (%)*** |  |  |  |  | ＜0.001 |
| Diastolic blood pressure≤76mmHg | 3488(75.3%) | 2683(73.3%) | 780(82.5%) | 25(89.3%) |  |
| Diastolic blood pressure＞76mmHg | 1144(24.7%) | 975(26.7%) | 166(17.5%) | 3(10.7%) |  |
| ***Waist circumference-factor (%)*** |  |  |  |  | 0.444 |
| Waist circumference≤108.8cm | 3479(75.1%) | 2757(75.4%) | 699(73.9%) | 23(82.1%) |  |
| Waist circumference＞108.8cm | 1153(24.9%) | 901(24.6%) | 247(26.1%) | 5(17.9%) |  |
| ***BMI-factor (%)*** |  |  |  |  | 0.313 |
| BMI≤32.6kg/m^2 | 3479(75.1%) | 2731(74.7%) | 725(76.6%) | 23(82.1%) |  |
| BMI＞32.6kg/m^2 | 1153(24.9%) | 927(25.3%) | 221(23.4%) | 5(17.9%) |  |
| ***Serum creatinine-factor (%)*** |  |  |  |  | ＜0.001 |
| Serum creatinine≤1mg/dL | 3647(78.7%) | 2938(80.3%) | 690(72.9%) | 19(67.9%) |  |
| Serum creatinine＞1mg/dL | 1153(21.3%) | 720(19.7%) | 256(27.1%) | 9(32.1%) |  |
| ***AL (median [IQR])*** | 2(1,4) | 2(1,4) | 2(1,4) | 3.00(1.00,3.75) | ＜0.001 |
| ***AL-factor (%)*** |  |  |  |  | ＜0.001 |
| Low AL | 2392(51.6%) | 1839(50.3%) | 541(57.2%) | 12(42.9%) |  |
| High AL | 2240(48.4%) | 1819(49.7%) | 405(42.8%) | 16(57.1%) |  |
| ***Depression score (median [IQR])*** | 3(2,6) | 3(2,6) | 3(2,6) | 3.5(2.0,7.0) | 0.822 |
| ***Depression score-factor (%)*** |  |  |  |  | 0.865 |
| Yes score≥10 | 630(13.6%) | 501(13.7%) | 126(13.3%) | 3(10.7%) |  |
| No score＜10 | 4002(86.4%) | 3157(86.3%) | 820(86.7%) | 25(89.3%) |  |
| ***Heart failure (%)*** | 144(3.1%) | 91(2.5%) | 49(5.2%) | 4(14.3%) | ＜0.001 |
| PIR: Poverty income ratio; LDL: Low-density lipoprotein; HDL: High-density lipoprotein; FBG: Fasting blood glucose; BMI: Body mass index; AL: Allostatic load; IQR: Interquartile range; CONUT: Controlling nutritional status | | | | | |

| **Supplementary Table 3 Baseline characteristics categorized by AL levels** | | | | |
| --- | --- | --- | --- | --- |
| **Variables** | **Overall N=4632 (100%)** | **Low AL N=2392(51.6%)** | **High AL N=2240(48.4%)** | **P-Value** |
| ***Age(median[IQR])*** | 46(32，62) | 46(28，56) | 53(39，65) | ＜0.001 |
| ***Sex(%)*** |  |  |  | ＜0.001 |
| Male | 2050(44.3%) | 895（37.4％） | 1155（51.6%） |  |
| Female | 2582(55.7%) | 1497（62.6%） | 1085（48.4%） |  |
| ***Race(%)*** |  |  |  | 0.276 |
| Mexican American | 697(15.0%) | 351（14.7%） | 346（15.4%） |  |
| Other Hispanic | 408(8.8%) | 211（8.8%） | 197（8.8%） |  |
| Non-Hispanic White | 2337(50.5%) | 1209（50.5%） | 1128（50.4%） |  |
| Non-Hispanic Black | 904(19.5%) | 442（18.5%） | 462（20.6%） |  |
| Other Race - Including Multi-Racial | 286(6.2%) | 179（7.5%） | 107（4.8%） |  |
| ***Education(%)*** |  |  |  | ＜0.001 |
| Less than high school | 1199(25.9%) | 531（22.2%） | 668（29.8%） |  |
| High school | 1103(23.8%) | 519（21.7%） | 584（26.1%） |  |
| More than high school | 2330(50.3%) | 1342（56.1%） | 988（44.1%） |  |
| ***PIR (%)*** |  |  |  | 0.001 |
| Low income | 3880(83.8%) | 1963（82.1%） | 1917（88.6%） |  |
| High income | 752(16.2%) | 429（17.9%） | 323（14.4%） |  |
| ***Smoking (%)*** | 2197(47.4%) | 1079（45.1%） | 1118（49.9%） | 0.001 |
| ***Hypertension(%)*** | 1629(35.2%) | 524（21.9%） | 1105（49.3%） | ＜0.001 |
| ***Coronary heart disease(%)*** | 176(3.8%) | 62（2.6%） | 114（5.1%） | ＜0.001 |
| ***Diabetes(%)*** | 550(11.9%) | 122（5.1%） | 428（19.1%） | ＜0.001 |
| ***Anemia(%)*** | 239(5.2%) | 121（5.1%） | 118（5.3%） | 0.748 |
| ***Heart attack(%)*** | 189(4.1%) | 62（2.6%） | 127（5.7%） | ＜0.001 |
| ***Stroke(%)*** | 193(4.2%) | 59（2.5%） | 134（6.0%） | ＜0.001 |
| ***Albumin(median[IQR])*** | 4.2(4.0,4.4) | 4.2(4.0,4.5) | 4.2(3.9,4.4) | ＜0.001 |
| ***Lymphocyte(median[IQR])*** | 1.9(1.6,2.4) | 1.9(1.5,2.3) | 2.000(1.600,2.475) | ＜0.001 |
| ***Total cholesterol(median[IQR])*** | 192(165,219) | 183(161,204) | 206(173,236) | ＜0.001 |
| ***CONUT(median[IQR])*** | 1(0,1) | 1(0,1) | 0(0,1) | ＜0.001 |
| ***CONUT-factor(%)*** |  |  |  | ＜0.001 |
| Normal | 3658(78.9%) | 1839（76.8%） | 1819（81.2%） |  |
| Mild | 946(20.4%) | 541（22.6%） | 405（18.1%） |  |
| Moderate | 28(0.7%) | 12（0.5%） | 16（0.7%） |  |
| ***Depression score(median[IQR])*** | 3(2,6) | 3(2,6) | 3(2,7) | ＜0.001 |
| ***Depression score-factor(%)*** |  |  |  | 0.002 |
| Yes score≥10 | 630(13.6%) | 289（12.1%） | 341（15.2%） |  |
| No score＜10 | 4002(86.4%) | 2103（87.9%） | 1899（84.8%） |  |
| ***Heart failure(%)*** | 144(3.1%) | 46（1.9%） | 98（4.3%） | ＜0.001 |
| PIR: Poverty income ratio; CONUT: Controlling nutritional status; CONUT: Controlling nutritional status; AL: Allostatic load; IQR: Interquartile range | | | | |

| **Supplementary Table 4 Threshold analysis of CONUT** | |
| --- | --- |
| **Outcome** | **the effect size, 95%CI, P value** |
| Model 1 Fitting model by standard linear regression | 0.01(0.005-0.015), 0 |
| Model 2 Fitting model by two-piecewise linear regression |  |
| Inflection point | 1.5 |
| <1.5 | -0.044(-0.07-0.018), 0.001 |
| >1.5 | 0.023(0.016-0.031), 0 |
| P for likelihood ratio test | <0.001 |
| CONUT: Controlling nutritional status; CI: Confidence interval | |
